# Retinol binding protein 4 and type 2 diabetes: from insulin resistance to pancreatic β-cell function

**DOI:** 10.1007/s12020-024-03777-5

**Published:** 2024-03-23

**Authors:** Jiahua Fan, Jinxing Hu

**Affiliations:** 1 State Key Laboratory of Respiratory Disease, Guangzhou Key Laboratory of Tuberculosis Research, Department of Clinical Nutrition, Guangzhou Chest Hospital, Institute of Tuberculosis, Guangzhou Medical University, Guangzhou, 510095 Guangdong PR China; 2 State Key Laboratory of Respiratory Disease, Guangzhou Key Laboratory of Tuberculosis Research, Department of Tuberculosis, Guangzhou Chest Hospital, Institute of Tuberculosis, Guangzhou Medical University, Guangzhou, 510095 Guangdong PR China

**Keywords:** Retinol binding protein 4, Type 2 diabetes, Insulin resistance, Pancreatic β-cell function

## Abstract

**Background and aim:**

Retinol binding protein 4 (RBP4) is an adipokine that has been explored as a key biomarker of type 2 diabetes mellitus (T2DM) in recent years. Researchers have conducted a series of experiments to understand the interplay between RBP4 and T2DM, including its role in insulin resistance and pancreatic β-cell function. The results of these studies indicate that RBP4 has a significant influence on T2DM and is considered a potential biomarker of T2DM. However, there have also been some controversies about the relationship between RBP4 levels and T2DM. In this review, we update and summarize recent studies focused on the relationship between RBP4 and T2DM and its role in insulin resistance and pancreatic β-cell function to clarify the existing controversy and provide evidence for future studies. We also assessed the potential therapeutic applications of RBP4 in treating T2DM.

**Methods:**

A narrative review.

**Results:**

Overall, there were significant associations between RBP4 levels, insulin resistance, pancreatic β-cell function, and T2DM.

**Conclusions:**

More mechanistic studies are needed to determine the role of RBP4 in the onset of T2DM, especially in terms of pancreatic β-cell function. In addition, further studies are required to evaluate the effects of drug intervention, lifestyle intervention, and bariatric surgery on RBP4 levels to control T2DM and the role of reducing RBP4 levels in improving insulin sensitivity and pancreatic β-cell function.

## Introduction

Diabetes mellitus (DM) is a chronic systemic metabolic disorder caused by a relative or absolute deficiency of insulin secretion and dysfunction of insulin action and mainly manifests as elevated blood glucose. According to the International Diabetes Federation (IDF), ~537 million adults worldwide were diagnosed with diabetes in 2021, and this number is expected to increase to 643 million by 2030 and 783 million by 2045 [1]. During this period, the world population is estimated to grow by 20%, while the number of people with diabetes is estimated to increase by 46% [1]. The prevalence of diabetes has risen sharply worldwide, and diabetes has become one of the essential chronic diseases that endangers human health [2]. China is the largest developing country. In the past 20 years, with social progress and economic development, the incidence of diabetes mellitus in China has also shown a continuous upwards trend. According to national survey data, the prevalence of diabetes mellitus in adults was estimated to be 2.6% in 2002 and increased sharply to 12.8% in 2017 [3]. Type 2 diabetes mellitus (T2DM) accounts for up to 90% of all diabetes mellitus cases [4]. The sharply increasing incidence of T2DM has led to substantial social and economic burdens on public health services in China. Although more than seventy medications have been approved for treating T2DM, only 50% of treated patients with T2DM achieve adequate glycemic control [5]. The lack of early and sensitive biomarkers of diabetes might be an important reason for the high incidence of diabetes. Therefore, identification of sensitive biomarkers of diabetes mellitus and the underlying mechanism are urgently needed, and these biomarkers have attracted increasing amounts of attention.

Retinol-binding protein 4 (RBP4) was originally known as the only transporter of vitamin A in circulation and is secreted mainly by hepatocytes and adipocytes [6, 7]; this protein is responsible for transporting retinol stored in the liver to target tissues to exert physiological effects. However, increasing numbers of studies have indicated that circulating RBP4 levels are associated with cardiometabolic diseases such as obesity [8], insulin resistance [9], hyperlipidaemia [10], hypertension [11], chronic liver diseases [12–14], atherosclerosis [15] and cardiovascular disease [16–20]. Moreover, studies have suggested that elevated serum RBP4 levels play a critical role in the development of T2DM and might be a novel marker of T2DM and play a role in its progression [8, 21]. Impaired insulin resistance and β-cell function are two major pathophysiological criteria for T2DM. Previous studies have shown that high RBP4 gene expression can induce insulin resistance and promote the development of T2DM [21]. In addition, many studies have shown that RBP4 levels are associated with islet β-cell function. In vitro, RBP4 intervention and gene overexpression significantly inhibited the insulin secretory function of β cells [22]. These results indicated that the association between RBP4 and T2DM is related to insulin resistance and impaired islet β-cell function. Therefore, an in-depth exploration of the relationship between RBP4 and T2DM and its mechanism of action has great significance for the prevention, diagnosis, treatment and prognosis estimation of T2DM.

However, there is controversy about the role of RBP4 as a marker of T2DM. Therefore, a critical review of those studies is needed. In addition, although previous reviews have comprehensively reviewed RBP4 levels in individuals with obesity and metabolic dysfunctions [23–26], for insulin resistance and secretion, the two main mechanisms of T2DM, studies have focused mainly on RBP4 and insulin resistance and have paid little attention to RBP4 and pancreatic β-cell function. Hence, there is a lack of reviews focused on investigating the effects of RBP4 on both insulin resistance and pancreatic β-cell function. In this review, we update and summarize recent studies examining the relationship between RBP4 and T2DM and the role of RBP4 in insulin resistance and pancreatic β-cell function. The literature search was based on PubMed listings up to 10 January 2024.

## Basic characteristics of RBP4

RBP4 is a member of the apolipoprotein family [24] and is the sole transporter for transporting circulating active vitamin A retinol metabolites [6, 7]. The RBP4 gene is located on chromosome 10 (10q23-q24) and is a region adjacent to elevated fasting blood glucose levels in Caucasian Europeans and T2DM in Mexican Americans [27, 28]. The gene encodes a protein of 201 amino acids with a molecular weight of 21 kDa [29, 30].

RBP4 is derived mainly from liver and adipose tissue [7, 31–33]. Nevertheless, its mRNA can be detected in several other tissues and anatomical structures, such as the kidney, brain, and lung [24]. The liver has the highest expression level of RBP4, and it has the largest storage of retinoids of any organ, accounting for nearly 80% of all retinoids in vivo [34]. The different sources of RBP4 may determine its influence on insulin resistance [35]. Studies have reported that the expression of RBP4 in liver and adipose tissue has different effects on insulin resistance. Overexpression of RBP4 in the liver does not lead to insulin resistance [36], but in adipose tissue, it does [37]. The origin of the increased circulating RBP4 levels is still controversial. Previously, researchers found that RBP4 was originally found in adipose tissue and was an adipokine. However, a recent study reported that RBP4 should be considered primarily a hepatokine rather than an adipokine, as circulating RBP4 derives exclusively from hepatocytes and is undetectable in the blood of mice with a hepatocyte-specific RBP4 knockout [7]. This finding was further supported by many other findings indicating that RBP4 is related to liver diseases [36–39]. In other words, although RBP4 is also expressed in tissues other than the liver, RBP4 secreted by these tissues will not enter the blood circulation under normal circumstances, except in some disease states. However, the reasons why hepatocyte-derived RBP4 reaches the circulation but not adipocyte-expressed RBP4 are currently unknown.

The concentration of RBP4 in blood, and therefore also retinol, is rather tightly regulated and is normally maintained at ~2–3 µmol/l in humans and ~1 µmol/l in mice, despite changes in the daily uptake of retinoids via the diet [40]. However, it should be noted that the RBP4 measurement method is important because not all commercially available kits reproducibly quantify RBP4; moreover, quantitative western blotting standardized to full-length RBP4 is a superior method for measuring RBP4 in serum [41]. Therefore, choosing a reliable method, such as quantitative Western blotting, is necessary when calculating the serum RBP4 level.

RBP4 has a specific binding site that can specifically bind to vitamin A to transport vitamin A from the liver to the target organ to realize the intracellular transport and metabolism of vitamin A, which helps vitamin A play a physiological role in the body [42]. In addition, the binding of RBP4 to retinol increases the stability and solubility of retinol, which functions to prevent nonspecific oxidation, reduce toxicity, and maintain physiological concentrations of retinol in the blood. When the body is deficient in vitamin A, retinyl esters are hydrolyzed and released as retinol, which binds to RBP4 and is secreted into the circulation [25]. In circulation, the retinol-RBP4 complex binds transthyretin (TTR) to form the tertiary retinol-RBP4-TTR complex. Binding to TTR stabilizes the retinol-RBP4 complex, decreases RBP4 loss by renal filtration, limits the free diffusion of RBP4 to other cells, and allows RBP4 to be recycled following retinol uptake into cells [17, 23]. After reaching the target cell, the retinol-RBP4 complex is separated from TTR and acts on the receptor stimulated by retinoic acid 6 (STRA6) on the target cell. Under the action of STRA6, retinol enters target cells and plays a related role in regulating cell metabolism, including maintaining dark vision and regulating immunity, cell differentiation, and embryo development [21, 43, 44].

## Associations between RBP4 levels and type 2 diabetes

RBP4 has long been known as a transporter of vitamin A. Although elevated serum and urine RBP4 levels are observed in T2DM patients, a causal relationship has not been demonstrated [45, 46]. In 2005, Yang et al. used DNA arrays and found that the expression of RBP4 is elevated in the adipose tissue of adipose-Glut4(^−/−^) mice; for the first time, they reported that RBP4 is a new adipokine in animal experiments and proved that RBP4 is closely related to insulin resistance and T2DM [21]. The following year, Graham et al. measured serum RBP4 levels in subjects with various clinical presentations and found that RBP4 is an adipocyte-secreted molecule that is elevated in the serum before the development of frank diabetes and appears to indicate insulin resistance [8]. An increasing number of epidemiological studies have shown that RBP4 is significantly associated with T2DM.

Cross-sectional studies have shown that RBP4 levels are significantly increased in individuals with impaired glucose tolerance (IGT), prediabetes, or T2DM and are related to various clinical parameters known to be associated with insulin resistance [47, 48], indicating a relationship between RBP4 levels and T2DM. Most importantly, prospective studies have consistently demonstrated a significant relationship between circulating RBP4 levels and the risk of incident T2DM in children, adults and elderly individuals [49–55], despite several reports of sex differences [50, 52, 53]. Specifically, as patients with prediabetes have a greater risk of developing diabetes, with a lifetime conversion rate to T2DM as high as 74% [56], several studies have focused on the early diabetes-predicting role of RBP4 in individuals with prediabetes. Meisinger et al. reported that RBP4 levels were associated with prediabetes, and the higher the RBP4 level was, the greater the risk of prediabetes [47]. In a cohort study, Liang et al. reported that after 6 years in 2091, elderly patients in the highest RBP4 subgroup also had an increased risk of T2DM compared to those in the lowest quartile of RBP4 [51]; moreover, the risk of T2DM increased with increasing RBP4 concentration when RBP4 levels were higher than 50 μg/ml. Our previous study revealed that serum RBP4 levels were significantly greater in patients with prediabetes than in patients without prediabetes, and serum RBP4 levels were associated with new T2DM after a 3-year follow-up [55]. These results from independent human cohorts revealed a significant association between RBP4 levels and T2DM in both the general and high-risk populations.

In addition, several large, community-based studies from the Framingham Heart Study linking elevated RBP4 levels to cardiometabolic risk also support the relationship between RBP4 levels and T2DM. Kaess et al. included 3658 participants in the Third Generation Framingham Heart Study cohort and reported that RBP4 was positively associated with insulin resistance and with distinct components of metabolic syndrome [57]. Zachariah et al. included 3777 participants who attended examination cycle 1 and reported that higher circulating levels of RBP4 and fetuin-A indicated future cardiometabolic risk [58]. Furthermore, 2034 participants who were not obese (body mass index <30) or not diabetic and had both parents were included in the Framingham offspring cohort. They found that parental diabetes was associated with lower adiponectin but higher RBP4 concentrations in offspring [59].

Moreover, genetic studies further support the inductive role of RBP4 in causing T2DM, as a gain-of-function human nucleotide polymorphism in the RBP4 promoter is correlated with an increased risk of T2DM [60]. For example, Kovacs et al. reported a role for RBP4 genetic variation in susceptibility to T2DM and insulin resistance [61]. Consistent with these findings, Hu et al. suggested that genetic variants in the RBP4 gene may be associated with the circulating RBP4 concentration and phenotypes related to glucose metabolism [62]. Munkhtulga et al. conducted a case‒control study involving 511 control and 281 type 2 diabetes patients and revealed that the RBP4–803GA promoter polymorphism influences the binding of hepatic nuclear factor 1alpha and is associated with increased serum RBP4 levels in diabetic patients [63]. Furthermore, van et al., using a prospective, population-based follow-up study, explored the associations of the -803GA polymorphism and retinol intake with T2DM risk. They reported that homozygous carriers of the -803A allele had an increased risk of T2DM by 1.83 times [60], which further demonstrated the significant relationship between RBP4 and T2DM.

In contrast to the positive associations reported for the human studies considered above, several studies found no association between RBP4 levels and T2DM. For example, a population study of men reported that RBP4 was associated with metabolic syndrome. However, RBP4 levels are still not associated with insulin resistance in men with T2DM or CVD [64]. Similarly, several studies have reported a significant association between RBP4 levels and the risk of T2DM, but this relationship exists only for women and not for men [50, 52, 53]. Additional studies have indicated that RBP4 mRNA expression in adipose tissue is sex-specific and regulated by leptin, while circulating RBP4 levels appear to be independent of RBP4 secretion in adipose tissue [65]. In addition, since RBP4 is mainly excreted through glomerular filtration, some studies have indicated that there may be no causal link between RBP4 and insulin resistance or T2DM and that the increased serum RBP4 levels in T2DM patients may be caused by reduced renal clearance efficiency [66–70]. In addition, the literature has reported that the ratio of circulating RBP4 to retinol is greater in patients with impaired glucose tolerance and T2DM than in healthy individuals. The RBP4/retinol ratio is a better indicator of glucose metabolism status than RBP4 is, suggesting that RBP4 (apo-RBP4) without bound retinol may be involved in abnormal glucose metabolism [71]. Furthermore, animal studies revealed that elevated levels of liver-derived RBP4 do not cause impaired glucose tolerance in mice, indicating that circulating RBP4 concentrations may not be responsible for impaired glucose homeostasis [36]. Taken together, the inconsistent conclusions about the relationship between RBP4 levels and T2DM might be partly due to differences in study designs, populations, sex differences, renal function, and RBP4 forms.

Nonetheless, it is worth noting that the results from meta-analyses supported the significant relationship between RBP4 levels and T2DM, as Tan et al. included published data from 8 studies comprising 8087 participants and concluded that high RBP4 levels were associated with an increased risk of T2DM [72]. In addition, other meta-analyses demonstrated that serum RBP4 concentrations in patients with T2DM are associated with diabetes-related renal dysfunction and diabetic retinopathy [73–75]. Han et al. investigated the association between RBP4 levels and diabetic retinopathy (DR) in patients with T2DM via a meta-analysis. They concluded that elevated RBP4 levels are strongly associated with DR and may play an essential role in its progression [73]. Park et al. conducted a meta-analysis of T2DM patients and concluded that the serum RBP4 concentration may be associated with diabetes-related renal dysfunction [74]. Zhang et al. further included 12 studies and showed that the levels of circulating RBP4 were significantly greater in both T2DM patients with microalbuminuria and T2DM patients with a decreased eGFR. Circulating RBP4 levels are positively correlated with the albumin-to-creatinine ratio (ACR) but negatively correlated with the eGFR [75]. In conclusion, all the results from the meta-analysis support the relationship between RBP4 levels and T2DM.

## The role of RBP4 in insulin resistance

Skeletal muscle and adipose tissue are the main sites of insulin-induced glucose uptake. When skeletal muscle is stimulated by insulin, GLUT4 in the cell membrane is transported from intracellular to external glucose. GLUT4 undergoes conformational changes, and glucose is transported and released into the cell for metabolism [76]. The membrane transport function of GLUT4 is a key rate-limiting step in glucose utilization in skeletal muscle and adipose tissue. GLUT4 expression is downregulated in adipose tissue but not in skeletal muscle in insulin-resistant patients. In addition, GLUT4 in adipose tissue is downregulated in individuals with obesity, T2DM, or metabolic syndrome [77]. In 2005, Yang et al. reported that selective knockout of GLUT4 in adipose tissues led to systemic insulin resistance and an increased risk of diabetes, and by using genetic microarray technology, they found that RBP4 levels were significantly greater in GLUT4 knockout mice in adipose tissue. Further experiments revealed that intraperitoneal injection of the RBP4 protein or an increase in the serum RBP4 concentration via transgenes led to systemic insulin resistance, while reducing the serum RBP4 concentration via drugs increased insulin activity [21]. These studies showed that RBP4 acts not only as a transporter of vitamin A but also as a novel cytokine involved in insulin resistance.

Since RBP4 was first reported to be closely related to insulin resistance, an increasing number of studies have focused on the association between circulating RBP4 levels and insulin resistance. A population-based study revealed that RBP4 levels were associated with insulin resistance in obese, impaired glucose, type 2 diabetic, nonobese, and nondiabetic populations [8]. Cross-sectional studies have shown that elevated circulating RBP4 levels are strongly and independently associated with insulin resistance in the adult general population [78–80], perimenopausal women [81], and individuals with T2DM [82]. A nested, retrospective cohort study provided evidence that increased RBP4 levels were associated with significantly greater odds of worsening insulin resistance and hypertriglyceridaemia in overweight, postpubertal, non-Hispanic black teenage participants [83]. Furthermore, a 10-year follow-up prospective study of 3445 school-aged children revealed that participants with higher childhood RBP4 levels had adverse cardiometabolic profiles at follow-up and that baseline RBP4 levels predicted hyperglycemia and insulin resistance in the 10-year follow-up phase, independent of baseline BMI [84]. Moreover, a genetic study also indicated a role for RBP4 genetic variation in susceptibility to T2DM and insulin resistance, possibly through an effect on RBP4 expression [61].

In contrast to the positive associations reported for the human studies considered above, some studies have reported an insignificant correlation between circulating RBP4 levels and insulin resistance [9, 64, 81, 85–91]. In addition to the small sample size and cross-sectional design, which are prone to research bias, several other factors may explain the discontent relationship between RBP4 levels and insulin resistance. First, Schweigert and colleagues suggested that microalbuminuria is a major determinant of elevated plasma RBP4 in T2DM patients [68]. Similarly, William reviewed several studies considering renal function and concluded that renal function might influence the relationship between RBP4 levels and IR [92]. Second, Thompson et al. reported that RBP4 is mainly produced by hepatocytes and that mouse adipocyte RBP4 is not a significant source of circulating RBP4, even in the setting of insulin resistance. Because hepatocytes are involved in both the uptake of dietary vitamin A by the liver and its mobilization from the liver [93–95] and because retinol is loaded into the newly synthesized apo-RBP4 in the endoplasmic reticulum, when retinol is unavailable, apo-RBP4 is not secreted and accumulates in the endoplasmic reticulum [95]. Thus, circulating levels of RBP4 might be influenced by vitamin A and liver function. Third, Kos et al. reported that circulating RBP4 levels were independent of AT-RBP4 secretion and further found that adipose AT-derived RBP4-mRNA expression is sex specific and regulated by leptin [65]. Fourth, in the review by Nono Nankam, obesity was not always linked to RBP4 levels or insulin resistance [23], and the authors concluded that the association between RBP4 levels and insulin resistance might reflect pathologies other than obesity, such as adverse fat distribution, adipose tissue dysfunction, dyslipidemia, and others. Correspondingly, the serum RBP4 concentration is correlated with insulin resistance independent of BMI, and elevated serum RBP4 concentrations have also been detected in lean insulin-resistant patients, nonobese individuals, and normal-weight individuals [8, 96–98]. Taken together, renal function, liver function, vitamin A and other adipokine levels, fat distribution, and adipose tissue dysfunction could also partly explain the inconsistent correlation between RBP4 levels and insulin resistance.

Despite these inconsistencies, the intervention study demonstrated that exercise decreased RBP4 levels in individuals with insulin resistance and that there was an inverse relationship between GLUT4 protein levels and serum RBP4 levels in adipose tissue [8]. Moreover, RBP4 levels in the serum reportedly decrease significantly in diabetic patients after treatment with drugs that improve insulin resistance, such as pioglitazone and rosiglitazone [99]. In addition, several studies have reported that treating insulin-resistant obese mice with retinoid fenretinide (a synthetic retinoid-based RBP4 antagonist) could reduce the serum RBP4 and total-body retinol levels and improve insulin sensitivity [21, 100, 101]. Thus, according to the studies above, we support the potential role of RBP4 in mediating or reflecting systemic insulin sensitivity and resistance.

**Fig. 1:**
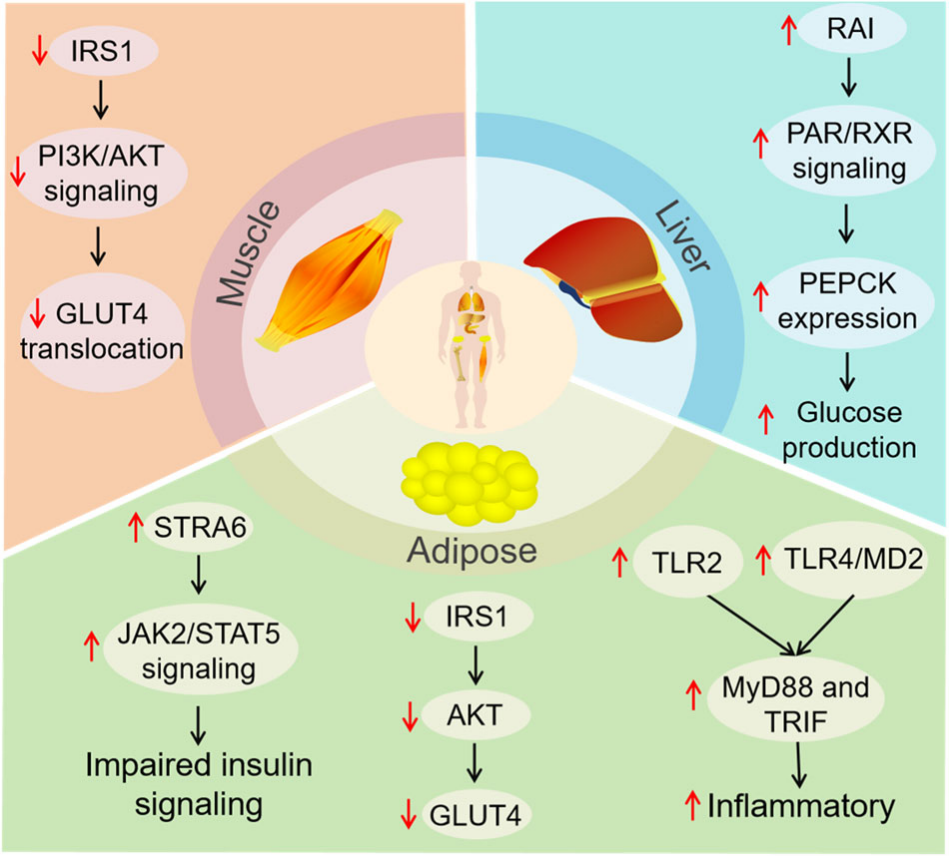
Molecular mechanisms of RBP4 in insulin resistance. In skeletal muscle, RBP4 inhibited the phosphorylation of IRS1, and the activation of PI3K resulted in impaired translocation of GLUT4 to the plasma membrane and induced insulin resistance. In the liver, RBP4 increases the production or alters the tissue metabolism of retinoic acid isomers, the active forms of retinol that interact with RARs and RXRs to regulate the expression of the retinoid-regulated gene PEPCK. The increase in PEPCK expression in hepatocytes induced baseline glucose production and reduced insulin-induced suppression of glucose production. In adipose tissue, RBP4 induces insulin resistance via the following mechanism: (left) RBP4 interacts with STRA6, after which activated JAK2/STAT5 signaling results in impaired insulin signaling; (middle) RBP4 inhibits IRS1, Akt1 and GLUT4. (right) RBP4 interacts with TLR2, TLR4/MD2 and the downstream pathways MyD88 and TRIF, leading to inflammatory infiltration in adipose tissue and inducing systemic insulin resistance. AKT protein kinase B, GLUT4 glucose transporter type 4, IRS1 insulin receptor substrate-1, JAK2 Janus kinase 2, MD2 myeloid differentiation protein 2, MyD88 myeloid differentiation primary response 88, PEPCK phosphoenolpyruvate carboxykinase, PI3K phosphoinositide 3-kinase, RAI retinoic acid isomers, RAR retinoic acid receptor, RBP4 retinol-binding protein 4, RXR retinoic acid-X receptor, STAT5 signal transducer and activator of transcription 5, TLR Toll-like receptor

The potential molecular mechanisms of RBP4 and insulin resistance are shown in Fig. 1. Yang et al. established an RBP4 gene knockout (RBP4^−/−^) and RBP4 overexpression (RBP4-Tg) mouse model to investigate the role of RBP4 in the development of insulin resistance. In skeletal muscle, they explored the effect of RBP4 on the insulin receptor PI3K and its downstream signaling pathway and found that the overexpression of the RBP4 gene in skeletal muscle tissue reduced the responsiveness of skeletal muscle to insulin by inhibiting insulin signaling pathway (IR/IRS/PI3K) activation and inducing insulin resistance [21]. In the liver, by increasing the production or altering the metabolism of retinoic acid isomers (RAIs), the active form of retinol that interacts with retinoic acid receptors (RARs) and retinoic acid-X receptors (RXRs), RBP4 induces the expression of the retinoid-regulated gene phosphoenolpyruvate carboxykinase (PEPCK) [21, 102]. As a result, the increase in PEPCK expression in hepatocytes induces baseline glucose production and reduces insulin-induced glucose production inhibition [21].

Adipose tissue is another important organ for the mechanistic link between RBP4 and insulin resistance. Researchers found that visceral fat RBP4 mRNA expression was significantly increased in obese and T2DM patients compared with that in control subjects, while subcutaneous fat RBP4 mRNA expression was not significantly different [103, 104]. Insulin-induced activation of insulin receptor substrate-1 (IRS1) and Akt1, as well as insulin-induced migration of GLUT4 to plasma membranes in adipocytes, are inhibited in mice treated with holo-RBP4 [105]. The binding of holo-RBP4 to STRA6, the cell surface receptor of RBP4, directly inhibits insulin signaling by activating the JAK2/STAT5/SOCS3 pathway [105, 106], leading to insulin resistance. Another study revealed that RBP4-mediated macrophage activation results in the blockade of insulin signaling in adipocytes. Mechanistically, RBP4 primes the NLRP3 inflammasome in macrophages through the TLR4/MD2 receptor complex and through TLR2 and the downstream pathways MyD88 and TRIF [107], releasing TNFα, IL-6, MCP-1, INF-γ, IL-1β, IL-2, IL-12, IL-8 and IL-10, which leads to the activation of the immune system, the promotion of the inflammatory state and the inhibition of insulin signaling. In addition, since obesity is also considered a low-level chronic inflammatory state characterized by the presence of inflammatory factors and infiltration of immune cells into adipocytes [108], many studies have reported that RBP4 is associated with a variety of inflammatory markers, including C-reactive protein, interleukin-6, tumor necrosis factor, and various cytokines [87, 109].

Furthermore, RBP4 may cause insulin resistance by activating both innate and adaptive immune responses. Moraes-Vieira et al. further reported that elevated RBP4 could trigger an adaptive immune response by activating innate immunity and that RBP4-overexpressing mice exhibited insulin resistance, glucose intolerance, increased expression and infiltration of adipose tissue macrophages and CD4+ T cells [110]. They found that in RBP4-overexpressing mice, adipose tissue CD206^+^ macrophages express proinflammatory markers and activate CD4+ T cells while maintaining alternatively activated macrophage markers. The above effects are mainly caused by the direct activation of adipose tissue antigen-presenting cells (APCs) through the c-Jun nitrogen terminal protein kinase (JNK)-dependent pathway. Further transfer of RBP4-activated APCs to normal mice causes an inflammatory response, insulin resistance, and glucose tolerance in mice [110], which suggests that RBP4 could, at least in part, activate adipose tissue APCs and thereby induce Th1 polarization and inflammation in adipose tissue in CD4+ T cells. A subsequent study demonstrated that RBP4 can activate antigen-presenting cells through the MyD 88-MAPK-NFκB pathway, leading to inflammatory infiltration in adipose tissue and inducing systemic insulin resistance [110, 111].

## RBP4 and pancreatic islet β-cell function

Pancreatic β-cell dysfunction plays a decisive role in the onset and progression of type 2 diabetes. Hyperglycemia does not occur unless β-cell function is no longer sufficient to overcome insulin resistance [112], and individuals with T2DM exhibit epigenetic alterations linked to mitochondrial dysfunction in pancreatic islets [113]. Although many studies have demonstrated that insulin resistance is one of the causes of diabetes, several population-based studies have shown that a positive correlation between RBP4 levels and the risk of T2DM still exists even when the insulin resistance indicator (HOMA-IR) is adjusted [55, 80], which indicates that RBP4 may increase the risk of diabetes through a pathway that does not completely overlap with insulin resistance.

Several studies have reported the relationship between RBP4 levels and pancreatic β-cell function. Several studies have reported an insignificant relationship between RBP4 levels and pancreatic β-cell function. Rasmus et al. reported no correlation between RBP4 levels and pancreatic β-cell function in young or elderly nondiabetic twins or in obese Caucasians without diabetes [114]. Another study reported a different relationship between RBP4 levels and pancreatic β-cell function in patients with different metabolic statuses. Ling et al. reported that the serum RBP4 concentration was positively correlated with glucose-stimulated insulin secretion in Chinese individuals with nonvisceral obesity and normal glucose tolerance (NGT); however, this association was not found in NGT subjects with visceral obesity or in subjects with T2DM [91].

However, animal studies have shown that vitamin A deficiency can lead to the loss of pancreatic β-cell enzymes and a decrease in insulin secretion, which may lead to hyperglycemia by inducing β-cell apoptosis [115, 116]. As the sole transporter of vitamin A in circulation, RBP4 is considered to be related to β-cell function. In fact, our previous study reported a U-shaped relationship between serum RBP4 levels and the risk of incident T2DM, and the RBP4-diabetes association was independent of insulin resistance, which indicates that RBP4 may increase the risk of T2DM through pathway(s) that do not largely overlap with insulin resistance and that RBP4 might be involved in the pathogenesis of β-cell dysfunction, which could be involved in the association between higher RBP4 levels and T2DM [55]. Similarly, additional studies have shown that there is a significant correlation between RBP4 levels and insulin secretion. In a population-based study, Yan et al. reported that RBP4 plasma levels were inversely correlated with the insulin secretion function of β-cells in female patients with non-alcoholic fatty liver disease [117]. Montserrat Broch et al. reported that the concentration of RBP4 was negatively associated with β-cell function in the whole population [90]. Chen et al. investigated the effect of the long noncoding RNA (lncRNA) PTGS2 on islet β-cell function and reported that PTGS2 can impair islet β-cell function by regulating miR-146a-5p and upregulating RBP4, which indirectly supports the relationship between RBP4 and islet β-cell function [118].

Moreover, it is worth noting that researchers have reported that antidiabetic drugs can decrease circulating RBP4 levels and improve β-cell function [119–121]. Pathophysiological conditions, such as obesity and non-alcoholic fatty liver disease, may impede the inconsistency of the conclusions of these studies. However, the methods used to assess β-cell function differ. Due to the inconsistency of β-cell function, insulin release is affected by many factors, and there is a lack of unified criteria for evaluating the insulin release function of β-cells in the population. In most of these studies, β-cell function was evaluated by HOMA-β, mean insulin levels, or the area under the curve for insulin from intravenous glucose-loading tests; these measures bypass the critical effect of intestine-derived incretin hormone on insulin secretion and are entirely nonphysiological.

**Fig. 2:**
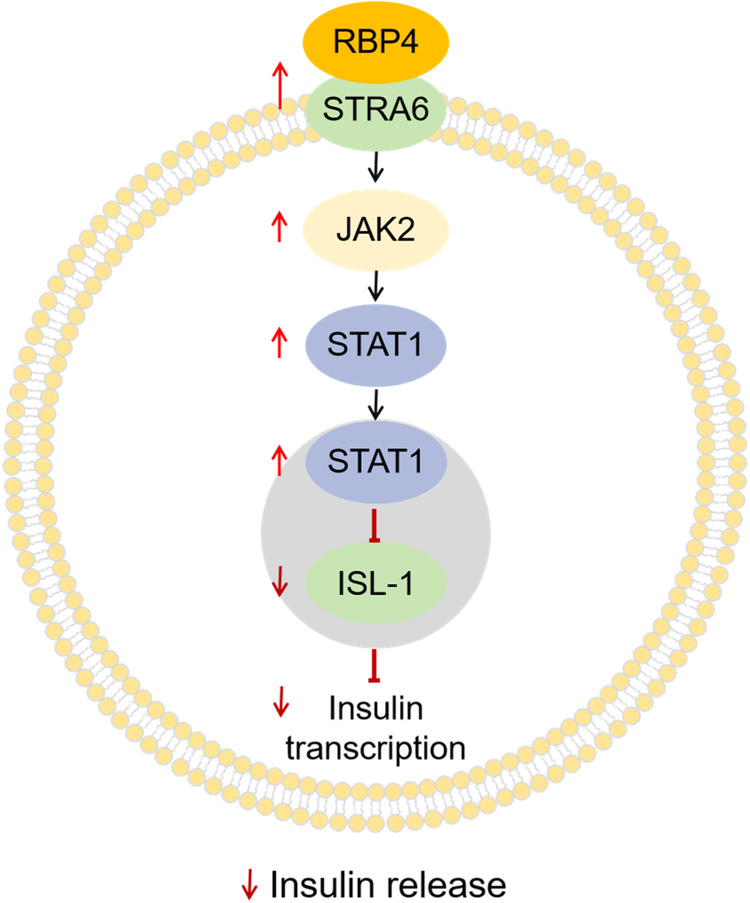
Molecular mechanisms of RBP4 in pancreatic β-cell function. In islets, STRA6 was specifically colocalized with insulin-positive β-cells. RBP4 can interact directly with STRA6 and upregulate its expression. RBP4/STRA6 activates the JAK2/STAT1 signaling pathway. The activation of STAT1 by RBP4 increased the binding ability of this gene to the promoter of Isl-1 and inhibited its expression, further decreasing insulin transcription and inhibiting insulin synthesis. STRA6 retinoic acid 6, JAK2 Janus kinase 2, STAT1 signal transducer and activator of transcription 1

Nevertheless, by evaluating β-cell function with both 2-h OGTT-derived indices and estimates derived from the oral minimal model test, which quantitatively simulates the complex process of glucose metabolism with a mathematical model and analogs of β-cell responsibility, Huang et al. first found that circulating RBP4 levels were negatively correlated with β-cell function across the spectrum of glycemia. In terms of mechanistic studies, they further investigated whether the increase in circulating RBP4 levels was inversely correlated with pancreatic β-cell function in db/db mice at different glycemic stages [22]. They found that RBP4 directly inhibited glucose-stimulated insulin secretion (GSIS) in primary isolated islets and INS-1E cells in a dose- and time-dependent manner. In RBP4 transgenic (RBP4-Tg) mice, the GSIS decreased dynamically and appeared as early as 8 weeks of age, resulting in impaired insulin sensitivity and glucose tolerance. There was a significant reduction in the number of islets isolated from RBP4-Tg mice with GSIS. Mechanistically, RBP4 can interact directly with STRA6, which is expressed in β cells and is the only known specific membrane receptor for RBP4, and its expression is upregulated. RBP4/STRA6 further activates the JAK2/STAT1 signaling pathway. Then, the activation of STAT1 increased the ability of the gene to bind to the promoter of Isl-1 and inhibited its expression, further decreasing insulin transcription and inhibiting insulin synthesis (Fig. 2). Moreover, reducing circulating RBP4 levels effectively reversed β-cell dysfunction and ameliorated hyperglycemia in db/db mice. These findings reveal a role for RBP4 in pancreatic β-cell dysfunction, which provides new insights into the diabetogenic role of RBP4.

## Approaches to lowering circulating RBP4 levels

The notion that elevated RBP4 levels in the circulation may contribute to T2DM has led to renewed interest in RBP4-lowering therapies, such as pharmacological treatment, lifestyle interventions (dietary weight loss and exercise training), and bariatric surgery.

According to pharmacological treatment studies, a reduction in the plasma RBP4 concentration occurs in response to different RBP4 antagonists, such as fenretinide, BPN-14136, and a high-affinity nonretinoid RBP4 ligand, which dissociates the RBP4-TTR complex. Fenretinide, a ligand of RBP4, has been suggested to reduce insulin resistance and associated disorders, such as obesity and fatty liver disease, by reducing the serum RBP4 concentration. Koh et al. reported a lower plasma RBP4 concentration in Fenretinide mice [101]. Kim et al. reviewed the potential therapeutic effects of RBP4 antagonists on the regulation of RBP4. They concluded that several RBP4 antagonists, especially BPN-14136, have demonstrated promising safety profiles and potential therapeutic benefits in animal studies. In addition, two RBP4 antagonists, tinlarebant (Belite Bio) and STG-001 (Stargazer), are currently undergoing clinical trials [122]. Circulating transthyretin (TTR) is a critical determinant of plasma RBP4 levels. Zemany et al. explored whether decreasing TTR levels with antisense oligonucleotides (ASOs) improves glucose metabolism and insulin sensitivity in obese individuals and reported that TTR-ASO treatment of mice with genetic or diet-induced obesity resulted in an 80–95% decrease in the circulating levels of TTR and RBP4 and improved insulin sensitivity in ob/ob mice and high-fat diet-fed mice as early as 2 weeks after treatment [123]. In addition, hypoglycemic drugs can also reduce RBP4 levels. Yang et al. reported that after 12 weeks of treatment with pioglitazone or metformin, pioglitazone was superior to metformin for decreasing RBP4 levels and HOMA-IR in patients with T2DM complicated with NAFLD [124]. Consistent with these findings, Lin et al. reported that the addition of pioglitazone could significantly lower the serum RBP4 and HOMA-IR values in T2DM patients who had been treated with metformin and/or sulfonylurea [125]. In addition, other studies have reported several potential treatments for T2DM involving reduced RBP4 levels [123, 126, 127].

Despite several lifestyle intervention studies reporting that lifestyle intervention leads to an insignificant change in RBP4 levels [128–130], additional studies support lifestyle intervention to reduce RBP4 levels and improve insulin sensitivity. Lee et al. reported a decrease in serum RBP4 levels with improved insulin sensitivity after a structuralized weight reduction program [131]. Reinehr et al. conducted a 1-year longitudinal follow-up study in a primary-care setting with 43 obese children and 19 lean children of the same age and sex based on exercise, behavior, and nutritional therapy and found that RBP4 levels were related to weight status and insulin resistance in both cross-sectional and longitudinal analyses, suggesting a relationship between RBP4 levels, obesity, and insulin resistance in children [132]. Marco-Benedí et al. conducted a 6-month weight loss intervention involving 73 obese individuals with T2DM who were randomized to follow one of two calorie-restricted diets, energy-restricted diets with standard (18% of total calories; SP) or high (35%) protein (HP) intake. They found that both calorie-restricted diets could lead to weight loss, insulin sensitivity improvement and RBP4 reduction. No differences in weight loss or RBP4 levels were found between diets, but HOMA-IR in the HP diet decreased 2-fold compared with that in the SP diet, and a greater decrease in insulin and glucose was detected at 6 months [133]. For the effect of exercise on RBP4 levels, Phillips and Cobbold reviewed the impact of exercise and RBP4 levels and concluded that the more intense the exercise is, the greater the positive effect on plasma RBP4 levels. In addition, they suggested that short-duration, high-intensity training may improve traditional T2DM risk markers and reduce RBP4 levels [134]. Later, Taghian et al. reported that aerobic exercise can decrease body composition, insulin resistance and RBP4 levels and can be beneficial for obese women’s health [135]. Moreover, Ghorbanian et al. concluded that a combined intervention of carbohydrate restriction with aerobic exercise, compared with carbohydrate restriction and aerobic exercise alone, improved RBP4 levels, HOMA-IR, and different body compositions and metabolic syndrome factors in middle-aged men with metabolic syndrome [136]. These findings suggest that lifestyle intervention could be a promising approach for preventing T2DM.

Bariatric surgery is regarded as a safe and effective way to treat morbid obesity. For bariatric surgery, several articles reported that the HOMA-IR index decreased after surgery; however, there was no correlation with RBP4 levels [137–139]. In contrast, more studies have demonstrated a marked decrease in RBP4 levels after bariatric surgery, which correlates with weight loss. Animal studies have shown that bariatric surgery leads to significantly lower HOMA-IR and RBP4 scores [140, 141]. A population-based study revealed that bariatric surgery led to considerable weight loss, improved insulin sensitivity, and significantly decreased RBP4 levels. Haider et al. included 33 morbidly obese patients with a BMI above 40 kg/m^2^ and 14 healthy subjects with a BMI less than 25 kg/m^2^ as controls. Fasting serum concentrations of RBP4 were measured before and 6 months after gastric banding surgery. They concluded that reductions in circulating RBP4 may improve insulin resistance in morbidly obese individuals after weight loss [142]. Oberbach et al. reported that bariatric surgery could lead to a significant reduction in RBP4 levels in children and adults with morbid obesity [143, 144]. Tschoner et al. investigated the relationship between RBP4 levels, visceral fat, and metabolic syndrome during pronounced weight loss after bariatric surgery [145]. They found a marked decrease in RBP4 levels after bariatric surgery, and the decrease in RBP4 levels correlated with a reduction in visceral fat mass. Consistent with these findings, Gómez-Ambrosi et al. demonstrated a decrease in RBP4 levels after surgery in subjects with weight loss and reduced body fat [146]. Mitterberger et al. further showed that bariatric surgery and long-term caloric restriction interventions improved insulin sensitivity and significantly lowered RBP4 levels, suggesting that lowering these RBP4 levels contributes to improved insulin sensitivity [147]. Therefore, reducing the circulating RBP4 concentration might be a potential way to improve insulin resistance and T2DM.

## Summary

Taken together, the results of animal experiments, prospective studies, meta-analyses, genetic studies and intervention studies support the significant associations between RBP4 levels, insulin resistance, pancreatic β-cell function, and T2DM. The discrepancies in findings from clinical studies might be due to differences in the studied populations, sex, metabolic status, renal function, and different methodological evaluations of RBP4 circulation and tissue levels and actions. Therefore, additional mechanistic studies are needed to determine the role of RBP4 in the onset of T2DM, especially in terms of pancreatic β-cell function. In addition, further studies are required to evaluate the effects of drug intervention, lifestyle intervention, and bariatric surgery on RBP4 levels to control T2DM and the role of reducing RBP4 levels in improving insulin sensitivity and pancreatic β-cell function.
